# Ursolic Acid Inhibits Adipogenesis in 3T3-L1 Adipocytes through LKB1/AMPK Pathway

**DOI:** 10.1371/journal.pone.0070135

**Published:** 2013-07-26

**Authors:** Yonghan He, Ying Li, Tiantian Zhao, Yanwen Wang, Changhao Sun

**Affiliations:** 1 Department of Nutrition and Food Hygiene, Public Health College, Harbin Medical University, Harbin, People's Republic of China; 2 Aquatic and Crop Resource Development, Life Sciences Branch, National Research Council Canada, Charlottetown, Prince Edward Island, Canada; 3 Department of Psychology, University of Toronto, Toronto, Ontario, Canada; 4 Department of Biomedical Sciences, University of Prince Edward Island, Charlottetown, Prince Edward Island, Canada; University of Santiago de Compostela School of Medicine – CIMUS, Spain

## Abstract

**Background:**

Ursolic acid (UA) is a triterpenoid compound with multiple biological functions. This compound has recently been reported to possess an anti-obesity effect; however, the mechanisms are less understood.

**Objective:**

As adipogenesis plays a critical role in obesity, the present study was conducted to investigate the effect of UA on adipogenesis and mechanisms of action in 3T3-L1 preadipocytes.

**Methods and Results:**

The 3T3-L1 preadipocytes were induced to differentiate in the presence or absence of UA for 6 days. The cells were determined for proliferation, differentiation, fat accumulation as well as the protein expressions of molecular targets that regulate or are involved in fatty acid synthesis and oxidation. The results demonstrated that ursolic acid at concentrations ranging from 2.5 µM to 10 µM dose-dependently attenuated adipogenesis, accompanied by reduced protein expression of CCAAT element binding protein β (C/EBP_β_), peroxisome proliferator-activated receptor γ (PPAR_γ_), CCAAT element binding protein α (C/EBP_α_) and sterol regulatory element binding protein 1c (SREBP-1c), respectively. Ursolic acid increased the phosphorylation of acetyl-CoA carboxylase (ACC) and protein expression of carnitine palmitoyltransferase 1 (CPT1), but decreased protein expression of fatty acid synthase (FAS) and fatty acid-binding protein 4 (FABP4). Ursolic acid increased the phosphorylation of AMP-activated protein kinase (AMPK) and protein expression of (silent mating type information regulation 2, homolog) 1 (Sirt1). Further studies demonstrated that the anti-adipogenic effect of UA was reversed by the AMPK siRNA, but not by the Sirt1 inhibitor nicotinamide. Liver kinase B1 (LKB1), the upstream kinase of AMPK, was upregulated by UA. When LKB1 was silenced with siRNA or the inhibitor radicicol, the effect of UA on AMPK activation was diminished.

**Conclusions:**

Ursolic acid inhibited 3T3-L1 preadipocyte differentiation and adipogenesis through the LKB1/AMPK pathway. There is potential to develop UA into a therapeutic agent for the prevention or treatment of obesity.

## Introduction

Obesity has become an epidemic in developed countries and also many developing countries. The rates of obesity and overweight have been continuing to grow in adults, and unfortunately that the situation has been worsening by penetrating into the child and adolescent population. In addition to morbidity, obesity is associated with many metabolic complications, including type-II diabetes, insulin resistance, hyperlipidemia, hypertension and coronary heart disease [1], [2]. These complications result in a considerably higher rate of mortality in obese than lean subjects.

Although a number of drugs have been developed and used to treat obese patients through regulating appetite, fat absorption and fat oxidation [3], [4], low efficacy and side effects are of great concerns and result in the withdraw of many anti-obesity drugs from market, leaving few drugs that can be prescribed [5], [6]. Various programs including lifestyle change and intensive exercise have been used to loose and help to control body weight, successful rate is marginal. It is still of demand to develop more efficacious and safer anti-obesity products/drugs. In recent years, numerous bioactive compounds in food items and plants, such as resveratrol [7], quercetin [8], and epigallocatechin gallate [9], have been explored for their potential anti-obesity activities. Ursolic acid is a natural pentacyclic triterpenoid, which is present in many different plants, fruits and herbs. Evidence from *in vitro* and *in vivo* studies suggests that UA possesses many nutritional and pharmacological functions, including anti-inflammatory [10], anti-oxidative [11], anti-mutagenic [12], anti-carcinogenic [13], hepatoprotective [14], anti-microbial [15], anti-atherosclerotic, and anti-hyperlipidemic effects [16]. Recent studies demonstrated that UA inhibited abdominal adiposity in mice fed a high-fat diet [17], [18]. It is reported that UA may reduce adiposity by enhancing lipolysis [19], [20] and/or inhibiting protein tyrosine phosphatase 1B (PTP1B) activity [21]. On the other hand, it is well known that adipogenesis plays a vital role in the development of obesity; however, information is lacking regarding whether and how UA modulates adipogenesis.

Adipogenesis is determined by multi-processes, which include preadipocyte proliferation, differentiation, and fatty acid oxidation and synthesis, and controlled by a number of molecular factors. Emerging evidence suggests that AMP-activated protein kinase (AMPK) functions as a sensor of cellular energy status. Once activated, it switches on the catabolic pathways and simultaneously switches off the ATP-consuming anabolic pathways [22]. AMPK provides an upstream signal of peroxisome proliferator-activated receptor γ (PPAR_γ_) and inhibits differentiation of preadipocytes into adipocytes [23], [24]. Furthermore, (silent mating type information regulation 2, homolog) 1 (Sirt1) is an NAD-dependent deacetylase that also serves as a master metabolic sensor, regulated by NAD^+^ concentration, and modulates cellular energy metabolism [25]. Sirt1 has been reported to inhibit adipogenesis in 3T3-L1 cells by repressing PPAR_γ_ [26] and is involved in the regulation of the number and function of adipocytes [27]. Therefore, the present study was conducted to determine the effect of UA on adipogenesis and mechanism of action, with the primary focus on the regulation of UA on the energy sensors AMPK and Sirt1 and further their downstream lipogenic targets in 3T3-L1 adipocytes.

## Materials and Methods

### Chemicals and reagents

Ursolic acid, nicotinamide, radicicol, insulin, 3-isobutyl-1-methylxanthine (IBMX), dexamethasone, propidium iodide, ribonuclease (Rnase) and protease inhibitor were purchased from Sigma (St. Louis, MO, USA). High glucose Dulbecco's modified Eagle's medium (DMEM) was from Mediatech, Inc. (Cellgro Mediatech, Inc. Manassas, VA). Fetal bovine serum (FBS) was from PAA Laboratories (Etobicoke, ON, Canada). Bovine calf serum (BCS) and adipogenesis assay kits were purchased from Cayman Chemical Company (Ann Arbor, Michigan, USA). Lipolysis assay kits were purchased from Zen-Bio, Inc. (Research Triangle Park, NC, USA). The BCA protein assay kit was from Thermo Scientific (San Jose, CA, USA). RIPA lysis buffer was from Millpore (MA, USA). Protein loading buffer was from Bio-Rad (Montreal, QC, Canada). Antibodies against sterol regulatory element binding protein 1c (SREBP-1c), pAMPK_α_ (Thr 172), AMPK_α_, fatty acid-binding protein 4 (FABP4), β-actin, carnitine palmitoyltransferase 1 (CPT1), acetyl-CoA carboxylase (ACC) and pACC were purchased from Santa Cruz Biotechnology, Inc. (Santa Cruz, CA). Antibodies against liver kinase B1 (LKB1), pLKB1, PPAR_γ_, CCAAT element binding protein α (C/EBP_α_) and C/EBP_β_ were from Cell Signaling Technology, Inc. (Beverly, Massachusetts, USA). Fatty acid synthase (FAS) antibody was from Novus Biologicals (Oakville, ON, Canada). BM chemiluminescence blotting substrate kit was from Roche Diagnosis (Laval, QC, Canada). LKB1 and AMPK small interfering RNAs (siRNAs), Opti-MEM, reduced serum medium and lipofectamine RNAiMAX were obtained from Life Technologies (Rockville, MD).

### Cell culture

3T3-L1 mouse embryo fibroblasts were obtained from American Type Culture Collection (Rockville, MD) and cultured in DMEM containing 10% BCS until confluent, and were then maintained in the same medium for an additional 2 days. Differentiation was induced 2 days post-confluence (day 0 of differentiation) by adding 0.5 mM IBMX, 1 µM dexamethasone, and 5 µg/mL insulin in DMEM with 10% FBS. After 2 days of incubation, culture medium was changed to fresh DMEM containing 10% FBS and 5 µg/mL insulin. Two days later, the medium was replaced with DMEM supplemented with 10% FBS and incubated for another two days. UA was added two days after confluence (day 0) and maintained during cell differentiation until the time when the cells were harvested for the following described tests.

### Cell viability

3T3-L1 preadipocytes were seeded onto 96-well plates at a density of 10^4^ cells/cm^2^ and differentiated with induction medium in the presence of 2.5 to 20 µM UA for 6 days, as described above. Cells were treated with MTT assay reagents (1 mg/mL) for 4 hours and the resulting formazan was solubilized in 150 µL dimethyl sulfoxide (DMSO) and further diluted 10 times with DMSO. The absorbance was measured at 570 nm on a Varioskan Flash spectral scanning multimode plate reader (Thermo Fisher Scientific, Waltham, MA).

### Preadipocyte proliferation

#### MTT assay

3T3-L1 preadipocytes were seeded at a low density of 3×10^3^ cells/cm^2^ in 96-well plates. The cells were grown in DMEM containing 10% BCS supplemented with increasing doses of UA for 24, 48, and 72 hours. MTT solution (1 mg/mL) was added to the medium and incubated for 4 hours. The purple formazan crystals were dissolved in 150 µL DMSO and the absorbance was read at 570 nm on the Varioskan Flash spectral scanning multimode plate reader as described above.

### Cell counting

3T3-L1 preadipocytes were seeded onto 24-well plates at a low density of 3×10^3^ cells/cm^2^. The cells were treated with different concentrations of UA for 24, 48, and 72 hours, respectively. The number of adherent cells was determined by direct counting using a hemocytometer and a mini-automated cell counter from Orflo (http://www.orflo.com) after being trypsinized. Three independent experiments were performed with triplicates in each experiment.

### Flow cytometric analysis of cell cycle and apoptosis

Three days post-confluence, 3T3-L1 preadipocytes were differentiated in the presence of 0, 2.5, 5, 10 µM ursolic acid for 24 hours. The cells were collected and fixed overnight with 70% ethanol at 4°C. Following wash with PBS, the cells were stained with propidium iodide solution containing 20 µg/ml of RNase for 30 minutes. Fluorescence-activated cell sorting (FACS) analysis was performed on a Becton–Dickinson FACScan system and data were analyzed using the Flowjo software (Version 7.6.1, Tree Star Software; San Carlos, CA, USA).

### Intracelluar lipid accumulation

3T3-L1 preadipocytes were seeded onto 96-well plates and were induced to differentiate in the presence or absence of ursolic acid as described above. Lipid accumulation was measured 6 days after the induction using a commercial adipogeneis assay kit. Briefly, the cells were fixed with lipid fixative solution, stained with Oil Red O and washed with distilled water. Intracellular lipids were extracted with the extraction solution included in the kit. The absorbance was read at 520 nm on the same plate reader as described above. Images were captured under a Nikon inverted microscope (ECLIPSE TE200) after washing with distilled water.

### Lipolysis assay

3T3-L1 preadipocytes were seeded onto 96-well plates and induced to differentiate 2 days after confluence. The differentiated Cells were treated with different concentrations of ursolic acid for 24 and 48 hours to detect cell viability as described above. Lipolysis was measured using a commercial 3T3-L1 lipolysis assay kit. Briefly, cells were washed twice with 200 µL wash buffer and then treated for 3 hours with 150 µL of 10 µM ursolic acid suspended in the assay buffer in a humidified incubator at 37°C. One hour prior to the assay, glycerol standards were prepared following the kit's instructions (Zen-Bio, Inc., Research Triangle Park, NC, USA). At the end of the incubation, 100 µL of medium was collected from each well and transferred to a new plate with addition of 100 µL of the glycerol reagent. After 15 minutes at room temperature, the absorbance was measured at the wavelength of 540 nm on the Varioskan Flash spectral scanning multimode plate reader

### Western blotting

After 6-day differentiation in the presence of UA, 3T3-L1 adipocytes were collected and lysed in ice-cold RIPA lysis buffer for 30 minutes. Protein content was determined using a BCA protein assay kit. Equal amount of protein for each sample was loaded and separated on a 10% SDS-PAGE. After electrophoretic separation, the proteins were transferred to a nitrocellulose filter using a semi-dry transfer cell (Bio-Rad) at 13 V for 1 hour, blocked with 5% skim milk for 1 hour at room temperature, and incubated with primary antibodies at 4°C overnight. The nitrocellulose filters were then incubated with horseradish-peroxidase conjugated secondary antibody at room temperature for 3 hours. Immunoreactive proteins were detected using the chemiluminescent ECL assay and quantified using the Molecular Imager software (Bio-Rad). C/EBP_β_ expression was determined 2 days after the induction of cell differentiation in the presence or absence of the indicated concentrations of UA. The expression of each protein was present as fold of the loading control, β-actin.

### LKB1 and AMPK gene silencing

3T3-L1 preadipocytes were transfected with LKB1 or AMPK siRNA oligonucleotide duplexe 1 day post the confluence with lipofectamine RNAiMax. Generally, 75 nM siRNA was transfected with 4 µl/well of lipofectamine in a 6-well plate. Lipofectamine RNAiMax and siRNA were individually diluted in 50 µL Opti-MEM medium, mixed, incubated for 20 minutes at room temperature, and then added to each well. The medium was removed and replaced with the induction medium in the presence or absence of UA after 24 hours of transfection. The effectiveness of siRNA knockdown was determined after 24 hours of transfection and on day 6 of cell differentiation, respectively, by measuring the expression of LKB1 or AMPK using the Western blotting.

### Statistical analysis

The statistical analyses were performed using SPSS 13.0 statistical program (version 13.01S; Beijing Stats Data Mining Co. Ltd). The treatment effect was determined using one-way ANOVA and followed by a post-hoc Dunnett's or Bonferroni's multiple comparisons test, where a P value less than 0.05 was considered significant. Data are presented as means ± SD.

## Results

### Effect of UA on fat cell viability

**Figure 1:**
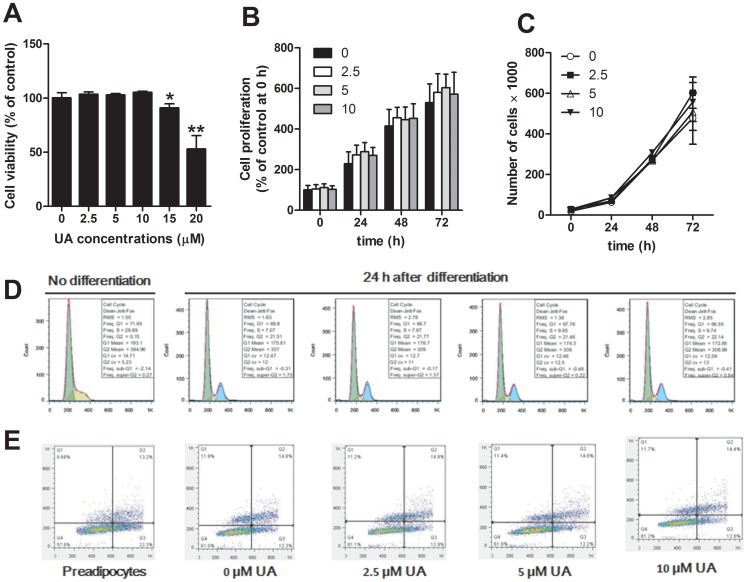
Effects of UA on the viability, proliferation, cell cycle and apotosis of 3T3-L1 preadipocytes. (A) 3T3-L1 preadipocytes were incubated in differentiation medium with or without UA. After 6 days, MTT reagent was added to the medium. After 4 hours of incubation, the medium was aspirated and 150 µL DMSO was added to each well. The absorbance was read at 570 nm. (B) 3T3-L1 preadipocytes were seeded onto 96-well plates at a density of 3×10^3^ cell/cm^2^ and treated with indicated concentrations of UA for 24, 48 and 72 hours, respectively. MTT assay was performed as described above to reflect the proliferation. (C) 3T3-L1 preadipocytes were seeded onto 24-well plates at a density of 3×10^3^ cell/cm^2^ and treated with different concentrations of UA for 24, 48 and 72 hours, respectively. The number of adherent cells was determined using an automatic cell counter. (D, E) Three days post-confluence, 3T3-L1 preadipocytes were differentiated in the presence or absence of 2.5, 5, 10 µM ursolic acid for 24 hours. The cells were collected and fixed overnight with 70% ethanol at 4°C, and stained with propidium iodide solution. Cell cycle and apoptosis rate were analyzed on a flow cytometry. Data are expressed as means ± SD (n = 3). * P<0.05 and ** P<0.001 vs. the control.

3T3-L1 preadipocytes were induced to differentiate in the presence of 0, 2.5, 5, 10, 15, or 20 µM of UA for 6 days. The MTT assay revealed that UA at concentrations of 2.5 to 10 µM did not affect cell viability while 15 µM was toxic (**Fig. 1A**). Therefore, the concentration range of 2.5–10 µM was chosen for further experiments. In mature adipocytes, 2.5 to 30 µM of UA did not affect cell viability after 24 hours of treatment whereas after 48 hours 20 µM and higher concentrations of UA were toxic (**Fig. S1A, B**).

### Effect of UA on preadipocyte proliferation, cell cycle and apoptosis

To investigate the effect of UA on preadipocyte proliferation, 3T3-L1 preadipocytes were grown in basal DMEM supplemented with different concentrations of UA for 24, 48 and 72 hours, respectively. As shown in **Fig. 1B** and **Fig. 1C**, neither microscopic counting nor MTT assay revealed any effect of UA on preadipocyte proliferation. The differentiation medium initiated cell cycle progression (**Fig. 1D**). The addition of different concentrations of UA did not affect cell cycle (**Fig. 1D**) nor did induce cell apoptosis (**Fig. 1E**).

### UA inhibits lipid accumulation and increases lipolysis in 3T3-L1 adipocytes

**Figure 2:**
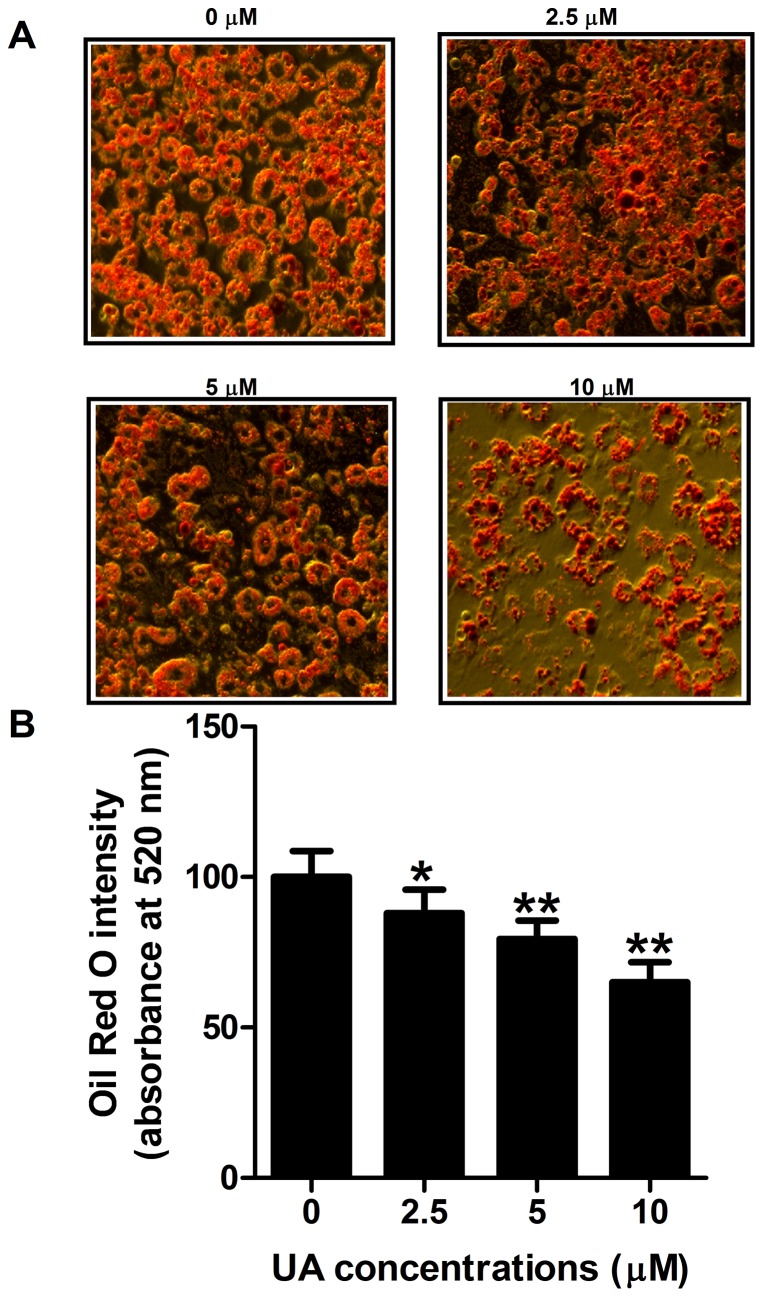
Effect of UA on lipid accumulation in 3T3-L1 adipocytes. (A) Post-confluent 3T3-L1 preadipocytes were induced to differentiate in the absence or presence of UA (added on day 0 of differentiation) for 6 days. The morphological changes associated with cell differentiation were photographed after Oil Red O staining. (B) Stained lipids were extracted and quantified by measuring absorbance at 520 nm. Data are expressed as means ± SD (n = 3). * P<0.05 and ** P<0.001 vs. the control.

3T3-L1 preadipocytes underwent morphological changes from the spindle-like features to round shape and accumulated intracellular lipids after adding the induction reagents. Ursolic acid at the doses of 2.5, 5, or 10 µM decreased intracellular fat content compared to the control as revealed by microscopic examination following Oil Red O staining in differentiated 3T3-L1 adipocytes (**Fig. 2A**). Consistently, the intracellular fat content was significantly reduced by UA at the concentrations ranging from 2.5 to 10 µM (**Fig. 2B**). Ursolic acid at the concentrations of 2.5, 5, and 10 µM reduced the lipid content in 3T3-L1 adipocytes by 10%, 19%, and 30%, respectively compared to the control. These findings suggest that UA was involved in the process of preadipocyte differentiation and adipogenesis. On the other hand, 10 µM UA significantly increased glycerol release compared to the control group (**Fig. S1C**), indicating that UA stimulated lipolysis in mature 3T3-L1 adipocytes.

### UA decreases protein expression of adipogenic transcription factors

**Figure 3:**
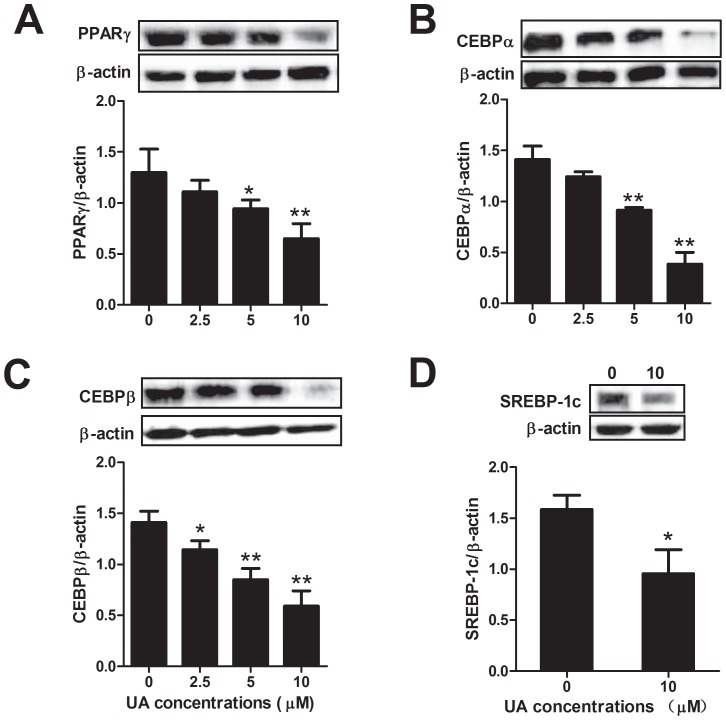
Effect of UA on the protein expression of differentiation-related transcriptional factors. (A)–(B) 3T3-L1 preadipocytes were incubated in differentiation medium without or with different concentrations of UA (added on day 0 of differentiation) for 6 days. The expression of PPAR_γ_ and C/EBP_α_ were assessed by Western blotting as described in the Materials and Methods. (C) The C/EBP_β_ expression was determined after preadipocytes were incubated in differentiation medium in the presence or absence of different concentrations of UA for 2 days. (D) The SREBP-1c expression was determined after preadipocytes were incubated in differentiation medium in the presence or absence of 10 µM UA for 6 days. Data are expressed as means ± SD (n = 3). *P<0.05 and **P<0.001 vs. the corresponding controls.

Differentiation of preadipocyte into adipocyte is tightly regulated by a sequential activation of several transcriptional factors, including C/EBP_β_, C/EBP_α_, PPAR_γ_ and SREBP-1c. Normally, C/EBP_β_ functions quickly following the induction of preadipocyte differentiation, followed by the expression of C/EBP_α_ and PPAR_γ_. As shown in **Fig. 3A–3C**, UA at 2.5 to 10 µM significantly decreased the expression of C/EBP_β_ on day 2 and subsequently inhibited the expressions of PPAR_γ_ and C/EBP_α_ on day 6 after the induction of differentiation. SREBP-1c expression was also significantly down-regulated by UA at 10 µM after 6 days (**Fig. 3D**).

### UA modulates the expression of lipogenic and fatty acid oxidation proteins

**Figure 4:**
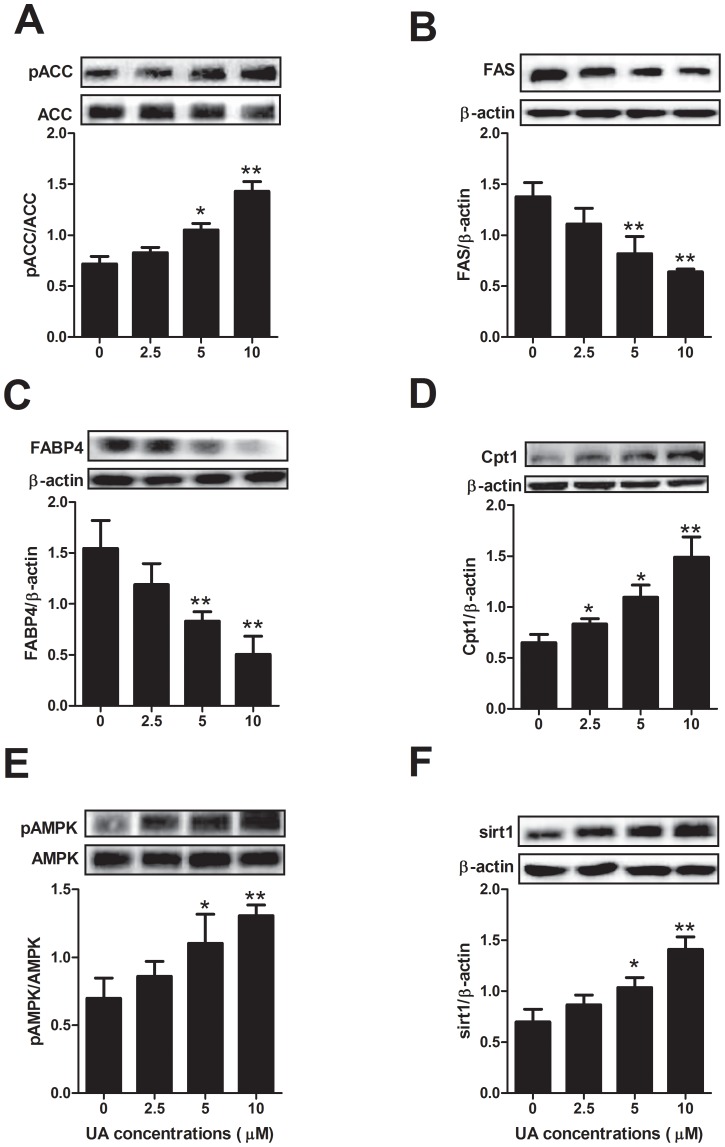
Effect of UA on the protein expression of lipogenic proteins, Cpt1, AMPK and Sirt1. 3T3-L1 preadipocytes were incubated in differentiation medium without or with different concentrations of UA (added on day 0 of differentiation) for 6 days. The expression of pACC, ACC, FAS, FABP4, Cpt1, pAMPK, AMPK and Sirt1 were assessed by Western blotting as described in the Materials and Methods. Data are expressed as means ± SD (n = 3). *P<0.05 and **P<0.001 vs. the corresponding controls.

Since the adipogenic transcription factors were down-regulated by UA, we further determined the expression and activation of their downstream protein targets such as ACC, FAS and FABP4, which are important adipogenic proteins involved in fatty acid and triacylglycerol biosyntheses. Ursolic acid inactivated ACC by increasing the phosphorylation of ACC (pACC) and reduced the expression of FAS and FABP4 at the concentrations of 5 and 10 µM, respectively (**Fig. 4A–C**). By contrast, UA at the concentrations of 2.5, 5 and 10 µM increased the protein expression of CPT-1 (**Fig. 4D**).

### UA regulates the expression or activation of energy sensors AMPK and Sirt1

AMPK and Sirt1 are two important regulators of preadipocyte differentiation and adipogenesis. In this study, UA at concentrations of 5 µM or 10 µM increased AMPK phosphorylation (pAMPK) and thus activation, while showing no effect on its total protein expression (**Fig. 4E**). Similarly, UA at 5 µM and 10 µM, respectively stimulated the protein expression of Sirt1 (**Fig. 4F**).

### Effect of AMPK and Sirt1 on adipogenic differentiation

**Figure 5:**
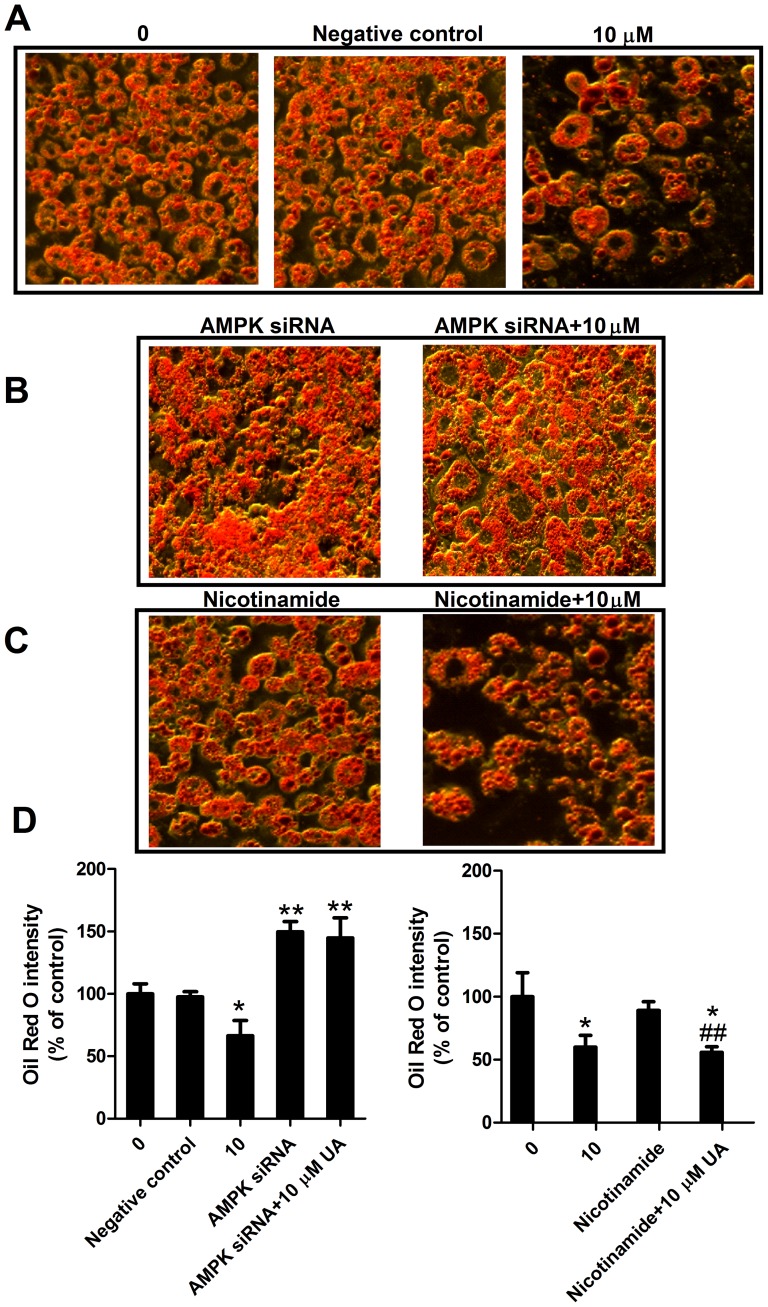
Effect of AMPK siRNA or Sirt1 inhibitor on lipid accumulation in 3T3-L1 adipocytes. (A–C) Post-confluent 3T3-L1 cells were induced for differentiation and treated with 10 µM UA (added on day 0 of differentiation) in the absence or presence of AMPK siRNA or 10 mM nicotinamide for 6 days. Images were captured using an inverted microscope after Oil Red O staining. (D) Stained lipids were extracted and quantified by measuring absorbance at 520 nm. Data are expressed as means ± SD (n = 3). *P<0.05 and **P<0.001 vs. the corresponding controls; ^#^P<0.05 and ^##^ P<0.001 vs. nicotinamide treated cells.

To further investigate the role of AMPK and Sirt1 in preadipocyte differentiation, both proteins were inhibited by AMPK siRNA and Sirt1 inhibitor nicotinamide, respectively. As shown in **Fig. S2A**, the AMPK siRNA significantly reduced AMPK protein expression, indicative of the effectiveness of AMPK siRNA transfection. The transfection of AMPK siRNA significantly increased lipid storage in differentiated 3T3-L1 cells as compared with the control (**Fig. 5B, D**). Ursolic acid at 10 µM significantly reduced fat content and strikingly, this effect was completely reversed by AMPK siRNA (**Fig. 5B, D**). Sirt1 inhibitor did not show a significant effect on intracellular fat accumulation as compared to the control nor did reverse the anti-adipogenic effect of UA (**Fig. 5C, D**).

### AMPK siRNA reversed the effect of UA on the AMPK downstream adipogenic targets

**Figure 6:**
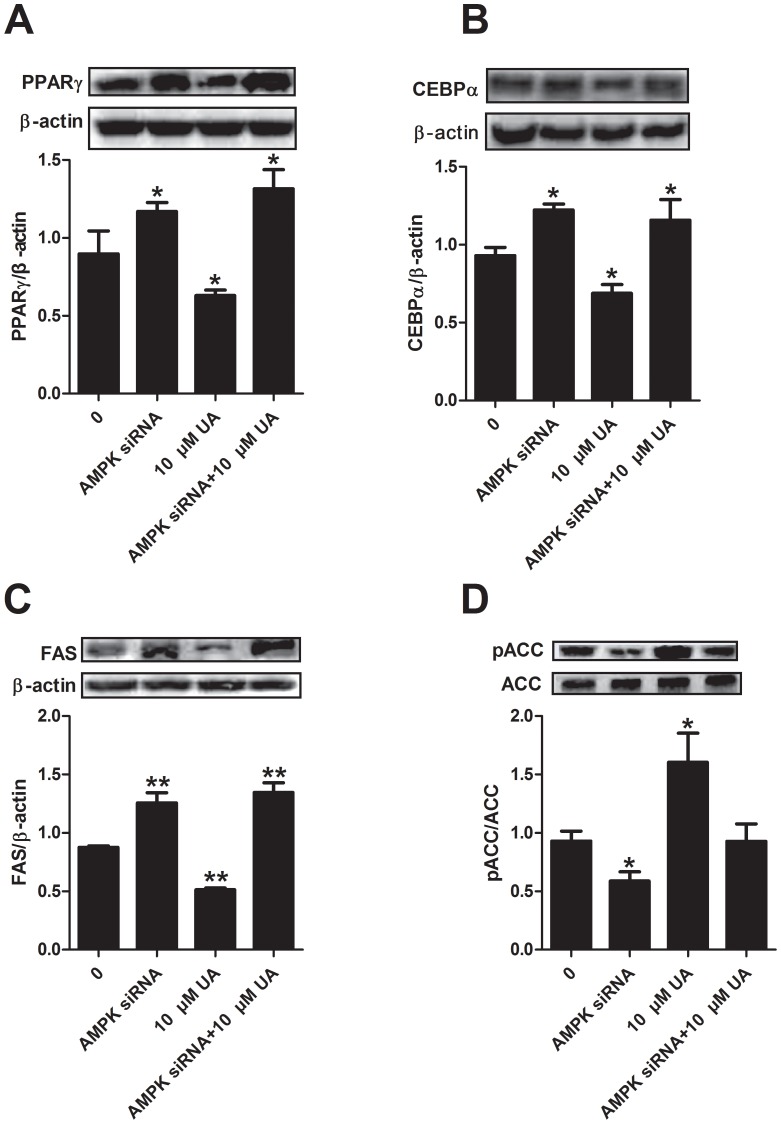
Effect of AMPK siRNA on the expression of differentiation transcriptional factors and lipogenic protein. Post-confluent 3T3-L1 cells were differentiated and treated with 10 µM UA for 6 days after the silencing of AMPK. PPAR_γ_, C/EBP_α_ and FAS, pACC and ACC expressions were assessed by Western blotting as described in the Materials and Methods. Data are expressed as means ± SD (n = 3). *P<0.05 and **P<0.001 vs. the corresponding controls.

To confirm whether UA modulates adipogenesis through AMPK, we detected the expression of adipogenic transcription factors, C/EBPα and PPARγ, and the key lipogenic gene FAS and ACC after cells were treated by AMPK siRNA. The application of AMPK siRNA diminished the effect of UA on PPARγ and C/EBPα (**Fig. 6A, B**). Consistently, the decreased expression of FAS by UA was reversed by the AMPK siRNA (**Fig. 6C**). The phosphorylation of ACC was inhibited by AMPK siRNA without affecting the expression of total ACC (**Fig. 6D**). It was demonstrated that UA regulated adipogenic process in 3T3-L1 preadipocytes through the AMPK pathway.

### LKB1 destabilizer or small interfering RNA diminishes the effect of UA on AMPK activation

**Figure 7:**
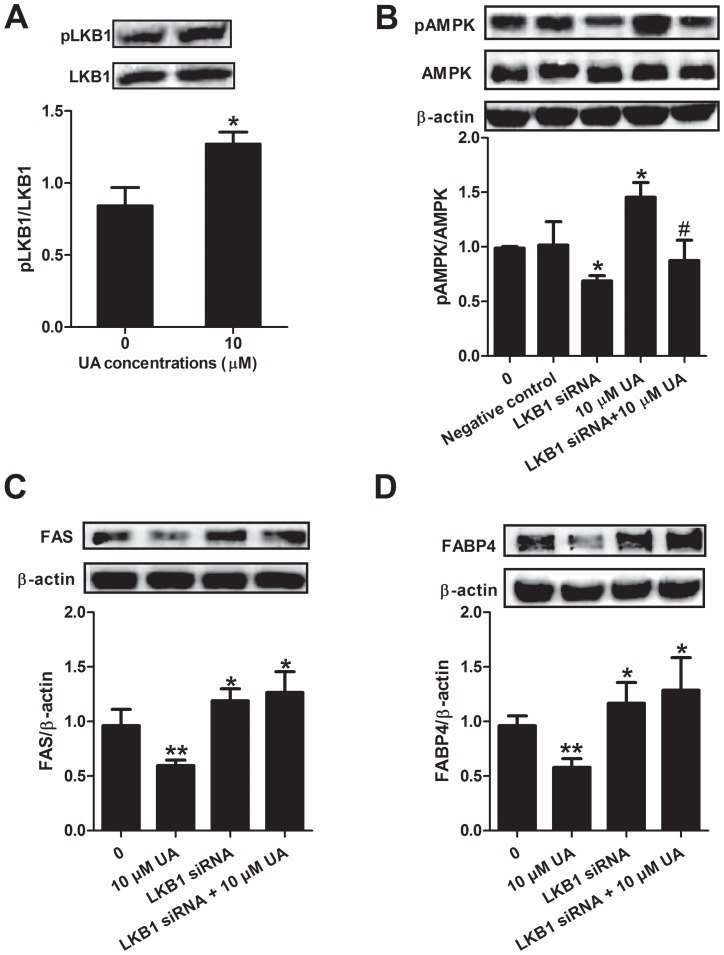
Effect of UA on the expression of LKB1, and the effect of LKB1 gene silencing on the expression of pAMPK, AMPK, FAS and FABP4. (A) 3T3-L1 preadipocytes were incubated in differentiation medium with or without 10 µM UA for 6 days. (B–D) Preadipocytes were transfected with LKB1 siRNA 1 day post confluence. Medium was removed and replaced with the differentiation-induction medium in the presence or absence of UA 24 hours after the transfection and maintained for differentiation for 6 days. pAMPK, AMPK, FAS and FABP4 expressions were assayed by Western blotting as described in the Materials and Methods. Data are expressed as means ± SD (n = 3). *P<0.05 and **P<0.001 vs. their corresponding controls; ^#^P<0.05 vs. UA treated cells.

To understand whether UA modulates AMPK activity in 3T3-L1 preadipocytes through its upstream regulator LKB1, we further investigated the effect of UA on the expression and activation of LKB1. It was observed that UA at 10 µM increased the LKB1 activity by increasing its phosphorylation (**Fig. 7** **A**). To further explore the role of LKB1 in the upregulation of UA on adipogenesis, LKB1 siRNA and its destabilizer radicicol were used, respectively. As shown in **Fig. S2B** and **2D**, total LKB1 expression was inhibited by either radicicol or LKB1 siRNA. Consequently, the increased phosphorylation of AMPK by UA was diminished when LKB1 siRNA or inhibitor was applied. (**Fig. 7B** **and Fig. S2C**), suggesting that UA activated AMPK via LKB1. To further evaluate the effect of LKB1 knockdown on adipogenesis in the presence of UA, we detected two key adipogenic proteins, FAS and FABP4. As shown in **Fig. 7C–7D**, LKB1 siRNA itself promoted the expression of FAS and FABP4 compared to the control group, which is similar to the effect of AMPK siRNA as described above. Furthermore, LKB1 siRNA abolished the inhibitory effect of 10 µM UA on FAS and FABP4.

## Discussion

Ursolic acid is present in many fruits and plants and the major component of several traditional medicine herbs. It is well known to that UA possess a wide range of biological functions [28]. Recent studies have demonstrated anti-obesity effect of UA; however, the mechanism of action is not well known. Adipogenesis and lipolysis determine, to a large degree, the adipose tissue mass. We previously demonstrated that UA stimulated lipolysis through the cAMP-dependent PKA pathway in primary-cultured rat adipocytes [20]. In this study, we also found that UA stimulated lipolysis in mature 3T3-L1 adipocytes. Most importantly, we report here, for the first time, that UA suppresses preadipocyte differentiation and adipogenesis through LKB1/AMPK pathway.

Adipose tissue is determined by the number and size of adipocytes. The increase in the number of adipocytes involves the proliferation of preadipocytes. Therefore, the growth and proliferation of preadipocytes has a profound implication in the development of obesity therapeutics or health products. Our results suggest UA did not affect preadipocyte number, cell cycle or apoptosis. However, UA significantly inhibited the differentiation of preadipocytes. After treatment with 10 µM of UA for 6 days, adipogenesis in differentiated 3T3-L1 cells was reduced by approximately 30%. Further studies demonstrated that several transcription factors were involved in the regulation of UA on adipogenic differentiation, including PPAR_γ_, C/EBP members and SREBP-1c [29], [30].

PPARγ functions through coordinating the expression of genes responsible for the establishment of mature adipocyte phenotype. The expression of PPAR_γ_ is sufficient to initiate and control adipogenesis in growing fibroblast cells by binding to PPAR response elements located in the promoter of target genes [31]. In addition, C/EBP proteins play critical roles in preadipocyte differentiation [32]. Among the C/EBP isoforms, C/EBP_β_ is responsible for the initial transcriptional activation of C/EBP_α_ gene [33], which is essential and sufficient to induce differentiation of 3T3-L1 preadipocytes [32]. Besides, SREBP-1c is a transcriptional factor that is involved in lipid metabolism [34], controls fatty acid synthase and is an additional regulator of adipogenesis. SREBP-1c promotes preadipocyte differentiation and gene expression associated with fatty acid metabolism [35]. In the current study, UA decreased C/EBP_β_ expression in 3T3-L1 preadipocytes during the first 48 hours after the induction of differentiation, and subsequently PPAR_γ_, C/EBP_α_ and SREBP-1c expressions on day 6, suggesting that UA inhibited preadipocyte differentiation through altering the expression of transcriptional factors involved at different differentiation stages. C/EBP_α_, PPAR_γ_ and SREBP-1c coordinately drive the expression of adipocyte-specific genes, such as FAS, ACC and FABP4, which determine the later stages of adipocyte differentiation and associated biosynthesis of fatty acids and triacylglycerols [36]. FABP4 is responsible for facilitating the influx of fatty acids across the plasma membrane and modulates the activity of enzymes involved in fatty acid metabolism [37]. ACC and FAS are the key enzymes controlling fatty acids synthesis [38], [39]. Our results revealed that these two proteins were inhibited by UA. Besides fatty acids synthesis, fatty acid oxidation is also critically important in the regulation of fat accumulation. CPT1 transports long chain fatty acids into mitochondria for beta oxidation and thus acts as a key regulatory enzyme in modulating fatty acid oxidation. The dose-dependent increase of CPT1 expression by UA suggests an increase of fatty acid oxidation. The findings of the current study suggest that UA can inhibit preadipocyte differentiation and adipogenesis through inhibiting the expression and/or activity of the adipogenic transcriptional factors and their target genes. Meanwhile, UA induced fatty acid oxidation could have also contributed to the inhibition of adipogenesis and reduction of fat accumulation by UA in differentiated 3T3-L1 preadipocytes.

Accumulating evidence suggests that AMPK and Sirt1 act as intracellular energy sensors and regulate energy metabolism through a concert process, and thus are of great interest in recent years as molecular targets in obesity research. AMPK directly modulates fatty acid synthesis and oxidation by altering the expression and activation of enzymes and proteins involved in fat metabolism. AMPK also regulates preadipocyte differentiation and adipogenesis [40]. It is reported that AMPK pathway is responsible for the inhibition of adipocyte differentiation by several natural compounds, such as apigenin [41], dioxinodehydroeckol [42], chitin [43], ginsenoside Rg3 [44], and epigallocatechin gallate [45]. Sirt1 inhibits adipogenesis by repressing PPAR_γ_ expression in cultured adipocytes [26] and decreases adipocyte formation during osteoblast differentiation of mesenchymal stem cells [46]. In this study, UA increased the phosphorylation and activation of AMPK and the protein expression of Sirt1 in a dose-dependent manner, indicating that UA might have inhibited preadipocyte proliferation and adipogenesis through AMPK and/or Sirt1. Further experiments showed that anti-adipogenesis of UA was abolished by AMPK siRNA but not by Sirt1 inhibitor. Consistently, the UA-induced reductions in the protein expression of transcription factors C/EBP_α_, PPAR_γ_ and their downstream protein FAS and phosphorylation of ACC, were interestingly reversed by blocking AMPK expression using AMPK siRNA.

LKB1 and calmodulin kinase kinase β are two main upstream kinases of AMPK [47]. LKB1 activates AMPK protein in adipose tissue [48], while the role of calmodulin kinase kinase β in AMPK activation is unclear. Therefore, we have moved a step further to determine the effect of UA on LKB1 expression. The observed increase in the phosphorylation and activition of LKB1 by UA suggests that UA might upregulate AMPK activity via LKB1. To verify this finding, LKB1 gene silencing and its destabilizer radicicol were employed to determine the effect of UA on AMPK activation in 3T3-L1 preadipocytes following the induction of differentiation. Surprisingly, the UA-induced AMPK phosphorylation and activation were abolished by either LKB1 gene silencing or LKB1 inhibition. Moreover, the UA induced reductions in the protein expression of FAS and FABP4 was reversed by blocking LKB1 expression using LKB1 siRNA. These results indicate the importance of LKB1 in the regulation of adipogenesis by UA. These results collectively demonstrated that UA stimulated LKB1 activity, resulting in the increase of AMPK phosphorylation and activation, and consequently the inhibition of AMPK downstream preadipocyte differentiation regulatory factors and their target lipogenic enzymes and proteins, and ultimately the decrease of adipogenesis.

Although we identified AMPK as the regulator through which UA exerted its anti-adipogenic effect, it is not possible to rule out other regulators involved in adipocyte differentiation and adipogenesis, such as Wnt signaling, GATA factors, and KLFs [49]. LKB1 is not the only upstream regulator of the AMPK signaling. TAK1 [50] and KSR2 [51], [52] have been shown to affect AMPK functions and, therefore, should be taken into considerations in future studies. Moreover, other proteins involved in adipocyte differentiation and adipogenesis await investigation. For example, Fsp27, an adipocyte-specific lipid droplet-associated protein, has been shown to play a role in the lipid droplet clustering and triglyceride accumulation [53]. The intracellular localization of C/EBP_β_ and the activity of PPARγ affect adipogenesis through a different way than their expressions. In addition, we used only 3T3-L1 adipocyte cell line to study the effect of UA on differentiation and adipogenesis. Our results and the above-mentioned items could be confirmed in other origins of adipocytes, such as human preadipocytes and pluripotent mesenchymal stem cells.

Encouragingly, we have conducted an *in vivo* study to evaluate the effects of UA on anti-obesity and improving the fatty liver in rats with high fat diet–induced obesity. The results revealed that UA at doses of 0.25% and 0.50% significantly reduced body weight and fat content, increased the fatty acid oxidation in skeletal muscle but without affecting the food intake (unpublished data). Thus, both *in vitro* and *in vivo* studies suggest the potential of UA to be an anti-obesity agent. Of course, the side effect of UA should be evaluated prior to the clinical application, especially in obese patients who are also diabetic as described in our studies [54].

Taken together, our results have demonstrated that UA inhibits 3T3-L1 preadipocyte differentiation and lipid accumulation by regulating the transcriptional factors and their downstream lipogenic targets via the activation of LKB1/AMPK pathway. UA is a promising naturally-occurring therapeutic agent for the prevention and treatment of obesity.

## Supporting Information

Figure S1 **Effect of UA on the viability and lipolysis in mature 3T3-L1.** (A–B) Differentiated 3T3-L1 adipocytes were incubated in different concentrations of UA for 24, 48 hours, respectively. MTT reagent was added to the medium. After 4 hours of incubation, the medium was aspirated and 150 µL DMSO was added to each well. The absorbance was read at 570 nm. (C) Mature adipocytes were treated with 10 µM ursolic acid for 3 hours. Lipolysis was quantified by measuring absorbance at 520 nm. Data are expressed as means ± SD (n = 3). * P<0.05 and ** P<0.001 vs. the control.
(TIF)

Figure S2 **Effectiveness of AMPK and LKB1 siRNA, and the effect of radicicol on the expression of LKB1, pAMPK and AMPK.** (A) and (D) 3T3-L1 preadipocytes were transfected with AMPK or LKB1 siRNA oligonucleotide duplexes 1 day post the confluence with lipofectamine RNAiMax. The effectiveness of siRNA knockdown after 24 hours of transfection and on day 6 of cell differentiation was determined by measuring the expression of AMPK and LKB1 using the Western blotting as described in the Materials and Methods. (B–C) Post-confluent 3T3-L1 cells were differentiated in the absence or presence of 5 µM radicicol for 6 days. The expression of LKB1, pAMPK, AMPK was measured using the Western blotting as described in the Materials and Methods. The bands of LKB1 and AMPK expression on day 6 of cell differentiation were shown. *P<0.05 and **P<0.001 vs. the control.
(TIF)
