# Supplementary figures and images for: Ursolic Acid Inhibits Adipogenesis in 3T3-L1 Adipocytes through LKB1/AMPK Pathway
Source: PLoS One. 2013 Jul 26;8(7):e70135. doi: 10.1371/journal.pone.0070135 (PMC3724828; doi:10.1371/journal.pone.0070135)

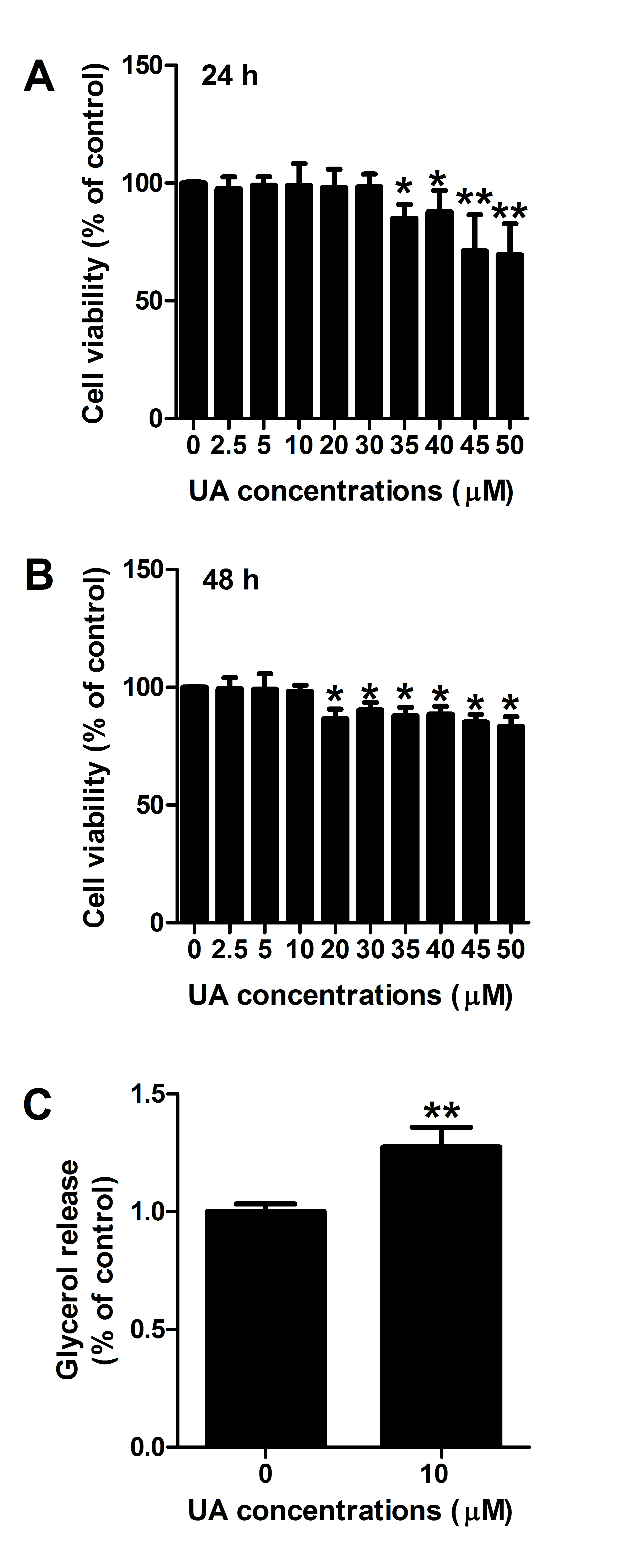

Supplement: Figure S1 — Effect of UA on the viability and lipolysis in mature 3T3-L1. (A–B) Differentiated 3T3-L1 adipocytes were incubated in different concentrations of UA for 24, 48 hours, respectively. MTT reagent was added to the medium. After 4 hours of incubation, the medium was aspirated and 150 µL DMSO was added to each well. The absorbance was read at 570 nm. (C) Mature adipocytes were treated with 10 µM ursolic acid for 3 hours. Lipolysis was quantified by measuring absorbance at 520 nm. Data are expressed as means ± SD (n = 3). * P<0.05 and ** P<0.001 vs. the control. (TIF) [file pone.0070135.s001.tif]

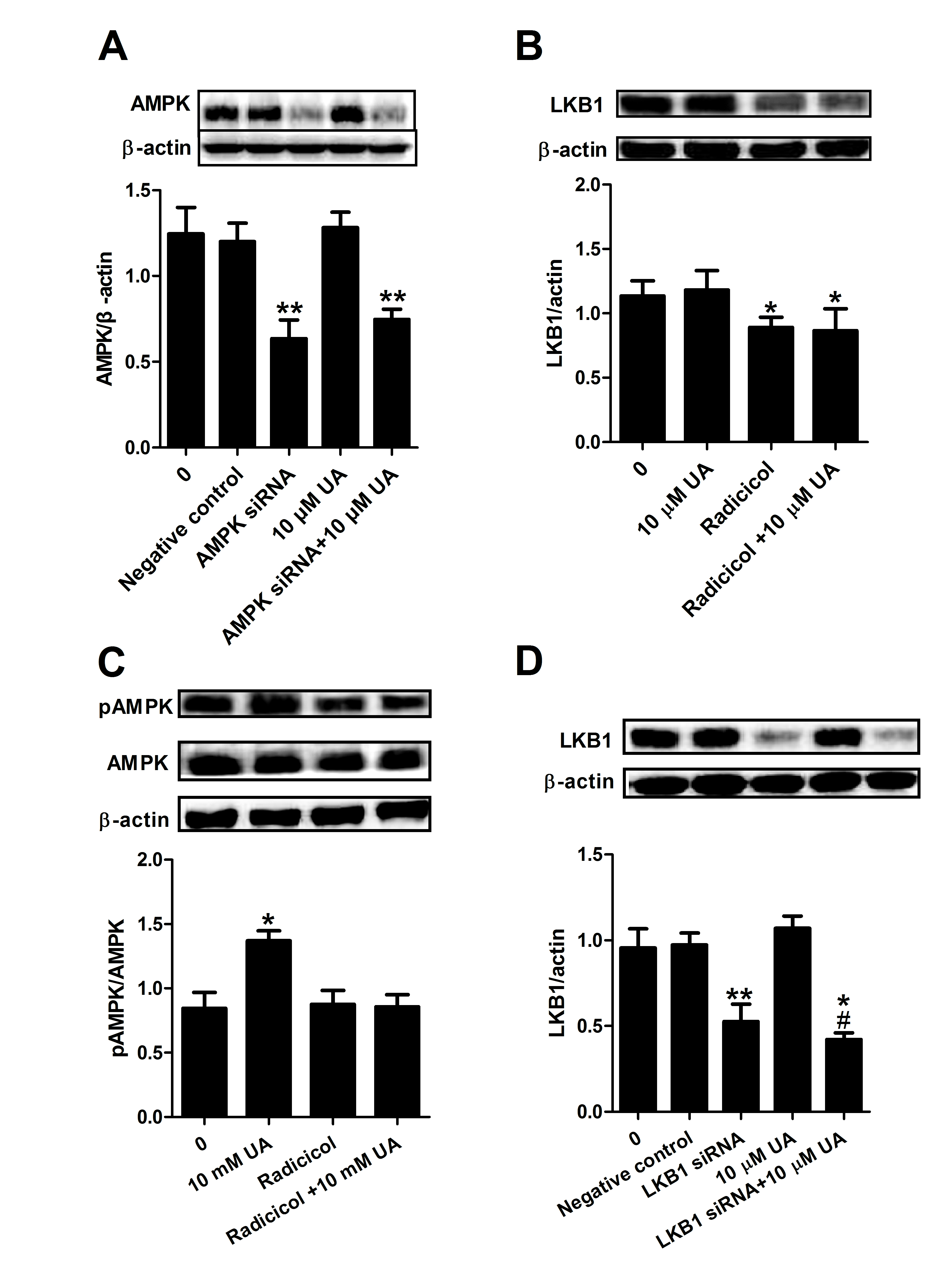

Supplement: Figure S2 — Effectiveness of AMPK and LKB1 siRNA, and the effect of radicicol on the expression of LKB1, pAMPK and AMPK. (A) and (D) 3T3-L1 preadipocytes were transfected with AMPK or LKB1 siRNA oligonucleotide duplexes 1 day post the confluence with lipofectamine RNAiMax. The effectiveness of siRNA knockdown after 24 hours of transfection and on day 6 of cell differentiation was determined by measuring the expression of AMPK and LKB1 using the Western blotting as described in the Materials and Methods. (B–C) Post-confluent 3T3-L1 cells were differentiated in the absence or presence of 5 µM radicicol for 6 days. The expression of LKB1, pAMPK, AMPK was measured using the Western blotting as described in the Materials and Methods. The bands of LKB1 and AMPK expression on day 6 of cell differentiation were shown. *P<0.05 and **P<0.001 vs. the control. (TIF) [file pone.0070135.s002.tif]
